# Improvement of the quantitation method for the *tdh*^+^* Vibrio parahaemolyticus* in molluscan shellfish based on most-probable- number, immunomagnetic separation, and loop-mediated isothermal amplification

**DOI:** 10.3389/fmicb.2015.00270

**Published:** 2015-04-09

**Authors:** Oscar Escalante-Maldonado, Ahmad Y. Kayali, Wataru Yamazaki, Varaporn Vuddhakul, Yoshitsugu Nakaguchi, Mitsuaki Nishibuchi

**Affiliations:** ^1^Department of Pathogenic Microbiology, Graduate School of Medicine, Kyoto UniversityKyoto, Japan; ^2^Department of Veterinary Science, Faculty of Agriculture, University of MiyazakiMiyazaki, Japan; ^3^Department of Microbiology, Faculty of Science, Prince of Songkla UniversityHat Yai, Thailand; ^4^Division of Human-Nature Dynamics, Center for Southeast Asian Studies, Kyoto UniversityKyoto, Japan

**Keywords:** *Vibrio parahaemolyticus*, most-probable-number, immunomagnetic separation, loop-mediated isothermal amplification, K antigen

## Abstract

*Vibrio parahaemolyticus* is a marine microorganism that can cause seafood-borne gastroenteritis in humans. The infection can be spread and has become a pandemic through the international trade of contaminated seafood. Strains carrying the *tdh* gene encoding the thermostable direct hemolysin (TDH) and/or the *trh* gene encoding the TDH-related hemolysin (TRH) are considered to be pathogenic with the former gene being the most frequently found in clinical strains. However, their distribution frequency in environmental isolates is below 1%. Thus, very sensitive methods are required for detection and quantitation of *tdh*^+^ strains in seafood. We previously reported a method to detect and quantify *tdh*^+^ *V. parahaemolyticus* in seafood. This method consists of three components: the most-probable-number (MPN), the immunomagnetic separation (IMS) targeting all established K antigens, and the loop-mediated isothermal amplification (LAMP) targeting the *tdh* gene. However, this method faces regional issues in tropical zones of the world. Technicians have difficulties in securing dependable reagents in high-temperature climates where we found MPN underestimation in samples having *tdh*^+^ strains as well as other microorganisms present at high concentrations. In the present study, we solved the underestimation problem associated with the salt polymyxin broth enrichment for the MPN component and with the immunomagnetic bead-target association for the IMS component. We also improved the supply and maintenance of the dependable reagents by introducing a dried reagent system to the LAMP component. The modified method is specific, sensitive, quick and easy and applicable regardless of the concentrations of *tdh*^+^ *V. parahaemolyticus*. Therefore, we conclude this modified method is useful in world tropical, sub-tropical, and temperate zones.

## Introduction

*Vibrio parahaemolyticus* inhabits estuarine and marine environments (Joseph et al., 1982). This bacterium thrives in high-temperature environments and thus it is prevalent in tropical areas year around and is found at lower concentrations only in summer in temperate regions.

*Vibrio parahaemolyticus* is the major cause of seafood-borne infections in the world (Raghunath, 2015). This bacterium can cause gastroenteritis in humans only when it propagates in the harvested seafood to the number exceeding the infectious dose when consumed by humans without proper cooking (Okuda et al., 1997a). A large number of *V. parahaemolyticus* cells distributed in the eutrophic coastal environments may accumulate in the digestive tract of molluscan bivalves because they filter-feed (Nishibuchi and Kaper, 1995; Manas et al., 2014). Therefore, molluscan shellfish accumulates *V. parahaemolyticus* at high concentrations and very frequently cause infection. However, not all strains are considered pathogenic. Only those possessing and expressing the gene (*tdh*) encoding the thermostable direct hemolysin (TDH) and/or the gene (*trh*) encoding the TDH-related hemolysin (TRH) are considered pathogenic (Nishibuchi and Kaper, 1985). Most clinical isolates of *V. parahaemolyticus* carry the* tdh* and *trh* genes, either alone or in combination; however, distribution of these genes in environmental isolates is usually low (1–2%; Nishibuchi and Kaper, 1995; Yamazaki et al., 2010) although some workers reported extremely frequent detection (up to 48%; Rodriguez-Castro et al., 2010; Gutierrez West Casandra et al., 2013).

The concentration of pathogenic *V. parahaemolyticus* can exceed a detectable level all the year around in the tropical zone but only exists at lower levels in the summer season in temperate zones. To not under-report pathogenic *V. parahaemolyticus*, sensitive methods have been devised for shellfish examination. These include the use of alkaline peptone water (APW) and salt polymyxin broth (SPB) as selective media in an enrichment procedure (Hara-Kudo et al., 2003); the immunomagnetic separation (IMS) technique targeting the K6 antigen shown to be useful in selective isolation of the O3:K6 pandemic strain of *V. parahaemolyticus* (Okuda et al., 1997b; Vuddhakul et al., 2000); and loop-mediated isothermal amplification (LAMP) reported to be more sensitive than conventional PCR (Yamazaki et al., 2010). Based on these reports, our group recently developed an most-probable-number (MPN) procedure for enumeration of *tdh*^+^ *V. parahaemolyticus* in shellfish samples where a PickPen-assisted IMS technique (hereinafter abbreviated simply as IMS) targeting as many as 69 established K antigens and a LAMP assay targeting the *tdh* gene were incorporated (Tanaka et al., 2014; hereinafter referred to as MPN-IMS-LAMP).

Experiments in southern Thailand show the MPN-IMS-LAMP performed well in general in detection and quantitation of *tdh*^+^ *V. parahaemolyticus* in shellfish products (Tanaka et al., 2014). However, there was a problem of underestimating MPN values because the study was conducted in a tropical environment where the total microbial population including target and non-target organisms is generally large; and, in such an environment, it is difficult to properly detect *tdh*^+^ *V. parahaemolyticus* (Vuddhakul et al., 2006). Overgrowth of non-target organism(s) and a failure in the IMS and the SPB enrichment are most likely responsible for the underestimation problem.

PickPen, an eight-channel intrasolution magnetic particle separation device enables a straight forward 96-well plate-based IMS procedure was successfully applied to increase the sensitivity and specificity of *Escherichia coli* O157:H7 detection in food (Nou et al., 2006). The IMS consists of two steps: the incubation of immunomagnetic beads (IMBs) with enriched culture containing the target bacterium and others (hereinafter referred to as IMB Incubation); and the washing step using the PickPen (hereinafter referred to as PickPen Operation) to remove non-targeted microbial population. The *E. coli* O157:H7 study demonstrated that constant shaking during the IMB Incubation could increase the efficiency of IMS (Nou et al., 2006). Our previous method employed incubation with intermittent mixing but not constant shaking (Tanaka et al., 2014). We also noticed loss of IMB during PickPen Operation suggesting improvement of this step in the IMS component. In this study we adopted the IMB Incubation with constant shaking and examined whether increased PickPen Operation time could further improve the efficiency of IMS.

Loop-mediated isothermal amplification allows one-step detection of gene amplification at a single temperature (Notomi et al., 2000) and it has been reported that LAMP is more simple and sensitive than the currently popular conventional PCR methods targeting the *tdh* and *trh* genes (Yamazaki et al., 2010). The conventional liquid LAMP reagent is practically inconvenient because refrigerated environment is recommended during storage, transportation, and operation; whereas the dried form does not require a refrigerated environment and therefore it can be used to detect pathogenic microorganisms even in tropical countries (Boehme et al., 2007; Mitarai et al., 2011). If the dried LAMP reagent is applicable to detection of the pathogenic genes of *V. parahaemolyticus*, it would be very useful for examination of seafood in various parts of the world.

In the present study, we attempted to improve the previously reported MPN-IMS-LAMP for quantitation of *tdh*^+^ *V. parahaemolyticus*. We evaluated the improvement by applying a series of modifications in the three components of the protocol: MPN, IMS, and LAMP. First, we evaluated two important factors (IMB incubation and PickPen Operation time) in IMS and we determined the benefits of the dried LAMP reagents for seafood analysis. Then, we compared the MPN values at three steps (after APW incubation, after IMS application, and after SPB enrichment). Finally, based on the results we present a new recommended MPN-IMS-LAMP method where the high sensitivity, MPN accuracy, and shellfish analysis applicability was determined.

## Materials and Methods

### Preparation of IMB

Immunomagnetic bead was prepared as previously described (Tanaka et al., 2014). In brief, magnetic beads were coated with antibodies partially purified with ammonium sulfate precipitation from polyvalent K antisera groups I–IX and monovalent anti-K70 to -K75 antibodies using commercially available *V. parahaemolyticus* K antisera from Denka Seiken Co., Ltd., Tokyo, Japan.

### Enrichment Procedures for Shellfish Samples

**FIGURE 1:**
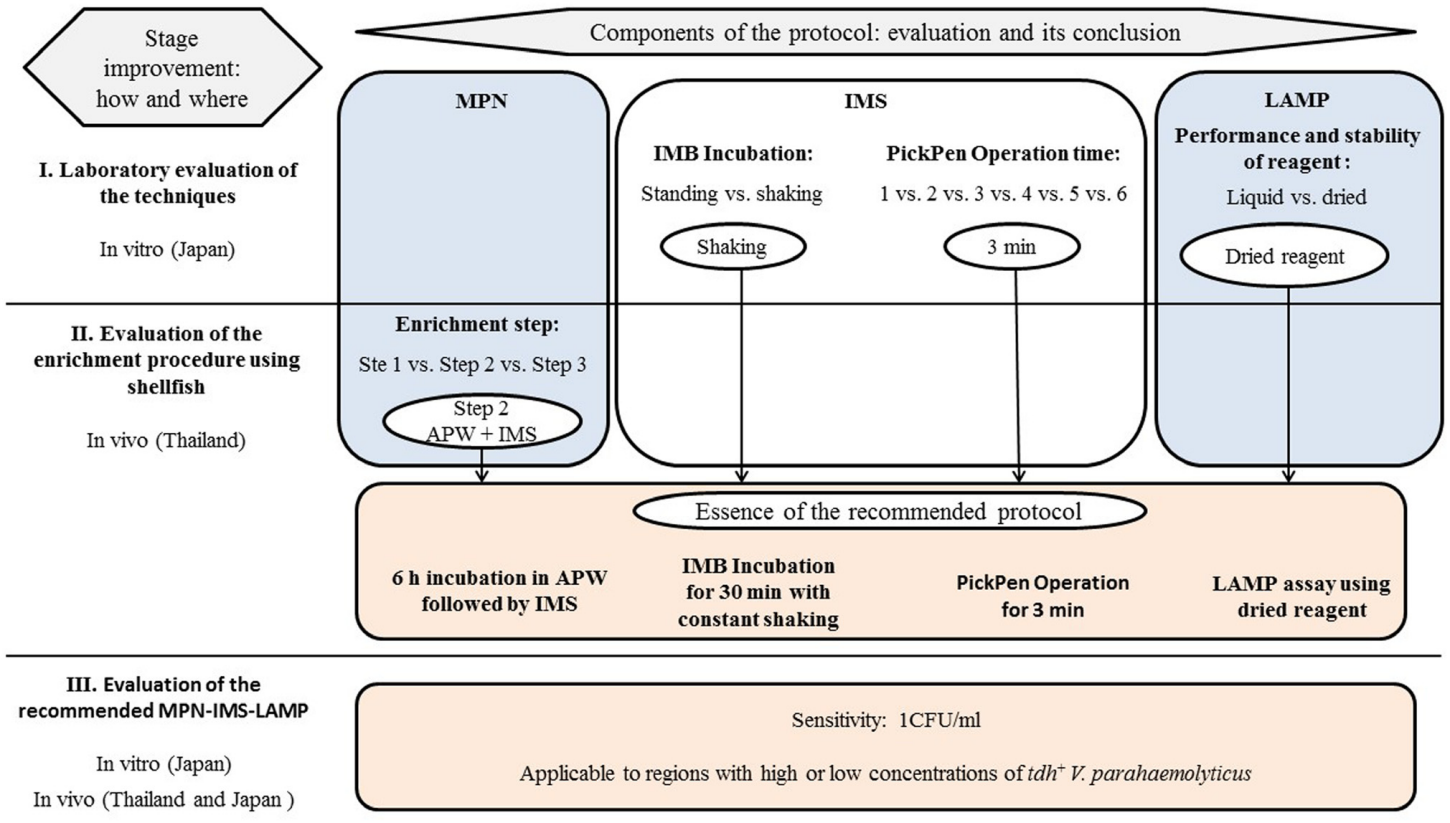
**MPN-IMS-LAMP protocol for quantitation of *tdh*^+^*V. parahaemolyticus* recommended in this study and the scheme of the comparative experiments used in this study**.

The shellfish sample was shucked and homogenized in a plastic bag. A three-tubes MPN dilution series was prepared as described in the U.S. Food and Drug Administration’s Bacteriological Analytical Manual (DePaola and Kaysner, 2004) with slight modifications (shown schematically in **Figure 1**).Briefly, a 25 g portion of the homogenate was added to 225 ml of APW (Nissui Pharmaceutical Co., Ltd., Tokyo, Japan). For determination of the MPN, the shellfish homogenate was diluted 10-fold as follows. Ten-ml of shellfish homogenate in APW was transferred to an empty tube, and subsequent 10-fold dilutions were prepared by transferring 1 ml aliquot to the tube containing 9 ml of APW in triplicate. The tubes were incubated at 37^∘^C for 6 h. The modified IMS was applied as described below (IMS subsection). The 1 ml suspension in PBS resulting from IMS was used for DNA template preparation followed by LAMP assay using dried reagent as described below. Finally, MPN values were determined.

### Immunomagnetic Separation

Immunomagnetic separation was performed as previously described (Nou et al., 2006; Tanaka et al., 2014) with modifications. Briefly, 1-ml aliquots of each culture were transferred to a well in a 96-well (2-ml capacity) titer plate. The cultures were mixed with 25 μl of the IMB were incubated on a shaker (140 rpm) at room temperature for 30 min. The subsequent bead washing and bead suspension steps were performed without changing the tips. The beads were captured with PickPen (BioControl Systems, Bellevue, WA, USA) by gently stirring the cultures with an up-and-down motion for 3 min. The captured beads were then washed twice by releasing into and recapturing from 1 ml of phosphate buffered saline (SIGMA-Aldrich Co., St. Louis, MO, USA) and were finally suspended in 1 ml of the same buffer.

### DNA Template Preparation

A 1-ml aliquot of a test culture was centrifuged at 15,000 rpm for 1 min, and the supernatant was discarded. The pellet was washed and suspended in 1 ml 0.85% NaCl solution, heated at 100^∘^C for 10 min, and immediately cooled on ice for 5 min. After centrifugation at 15,000 rpm for 5 min, the supernatant was transferred to a new tube and was stored at -20^∘^C until used.

### LAMP Assay Using Dried Reagent

The microtubes containing the dried reagent were taken from a commercially available kit for detection of Influenza virus (Loopamp Type A Influenza detection kit, Eiken Chemical Co., Ltd., Tokyo, Japan). The microtubes were transported, stored, and rehydrated at room temperature. Five μl of DNA template solution, 1.3 μl of *tdh* LAMP primer set (Yamazaki et al., 2010) and 23.7 μl of distilled water were added to make a final volume of 30 μl per reaction were added to the microtube. The cap was tightly secured and the microtube was inverted for 3 min in order to rehydrate the reagent which is located in the cap of the tube. The tube was heated at 65^∘^C for 1 h (reaction) and at 80^∘^C for 5 min (enzyme inactivation). Results of the reaction was judged using the visual system by the color change from brown to green in the reaction solution.

### Effect of PickPen Operation Time

*Vibrio parahaemolyticus* O3:K6 strain VP81 originally isolated from a fecal sample of a patient with diarrhea in India (Matsumoto et al., 2000) was used in this study. The strain was grown in 5 ml Luria broth (SIGMA-Aldrich Co.) at 37^∘^C with shaking at 160 rpm for 18 h. Serial 10-fold dilutions of the culture were made in sterile saline. Based on our preliminary experiment (data not shown), to best evaluate the effect of the PickPen Operation time, a concentration of 10^3^ CFU/ml was used for this experiment. Six sets of 25 μl of IMB mixed with the diluted culture were prepared and incubated for 30 min with shaking as explained above. The IMB in each set was then washed with different PickPen Operation times (1, 2, 3, 4, 5, or 6 min). After the IMB capture, the remaining supernatant as well as the diluted culture without IMS treatment were plated onto thiosulfate citrate bile salt sucrose agar (TCBS, Eiken Chemical Co.) and incubated at 37^∘^C for 24 h and colonies were counted. The capture efficiency (CE) was calculated using the following equation: CE (%) = (Co-Ca)/Co x 100%, where Co is the total CFU/ml present in the sample, and Ca is the CFU/ml not bound to IMB (Zeng et al., 2014).

### Comparison of Liquid and Dried LAMP Reagent

To compare the performance between liquid reagent (DNA amplification kit, Eiken Chemical Co.) and the dried reagent (contained in the microtubes provided in the Loopamp Type A Influenza detection kit), the DNA templates prepared from 14 *V. parahaemolyticus* reference strains were used. LAMP reaction with liquid reagent was performed according to the manufacture’s instruction. LAMP reaction with dried reagent is described above. The LAMP primer sets for the detection of *tdh*, *trh*1 and *trh*2 genes were used to conduct four LAMP assays targeting the *tdh*, *trh*1, *trh*2 and *tdh* plus *trh*2 genes as previously reported (Yamazaki et al., 2010). We judged the results using a turbidimetric system 1 h after the beginning of the reaction using the Loopamp EXIA LA-320A (Eiken Chemical Co.). Results were judged using the visual system after 1 h by a change in the color of the reaction solution. Finally, to assess the utility of the dried LAMP reagent in tropical countries, we tested its stability at high-temperatures. The reagents were exposed to temperatures of 30, 40, 50, and 60^∘^C for 15 days; and after, a *tdh* LAMP assay using a standard *tdh*^+^ *V. parahaemolyticus* strain was performed (Yamazaki et al., 2010).

### Shellfish Samples

Twenty eight samples of shellfish harvested in Thailand consisting of 12 bloody clams (*Anadara granosa*), 12 hard clams (*Meretrix lusoria*), and four green mussels (*Perna viridis*) were purchased at a local morning market in Hat Yai, Thailand, during April and May 2014. The shellfish samples were transported to the Prince of Songkla University, Hat Yai, Thailand at room temperature (∼30^∘^C) and were processed within 1 h of purchase. Sixteen samples of shellfish harvested in Japan consisting of 12 Japanese littlenecks (*Ruditapes philippinarum*) and four hard clams were obtained at Osaka Municipal Wholesale Market, Osaka, Japan during August and September 2014. These shellfish samples were transported to the laboratory at Kyoto University at room temperature (∼25^∘^C) and processed within 2 h of purchase.

### Modified Protocol for Comparative Experiments

A minor modification was employed in the experiments in southern Thailand for comparison. Samples were processed as described above after up to 6 h incubation in APW at 37^∘^C (**Figure 1**, Step 1). After IMS application (**Figure 1**, Step 2), 500 μl of 1-ml bead suspension was inoculated into 4 ml of SPB (Nissui Pharmaceutical Co., Ltd.) and was incubated at 37^∘^C for 18 h (**Figure 1**, Step 3). One-milliliter aliquots from each culture tube at Steps 1 and 3 and 500 μl of the bead suspension at Step 2 were used for DNA template preparation. LAMP using the liquid reagent was employed in these experiments.

### Sensitivity of the Recommended Protocol

Two-hundred-fifty ml of the Japanese littleneck sample suspension in APW was prepared by inoculation of *tdh*^+^ *V. parahaemolyticus* VP81 in the mid log phase at known concentrations (0.1, 1.0, 10.0 CFU/ml). These VP81 suspensions were examined for MPN of *tdh*^+^ *V. parahaemolyticus* using the protocol recommended in this study.

### Statistical Analysis

All experiments were performed in triplicate. The means and standard deviations of all collected data were calculated for every triplicate group. A student’s *t*-test was used for statistical analysis between two groups. A *p*-value ≤ 0.05 was considered statistically significant.

## Results

### The Strategy Used to Improve the Previous Quantitation Method

**FIGURE 2:**
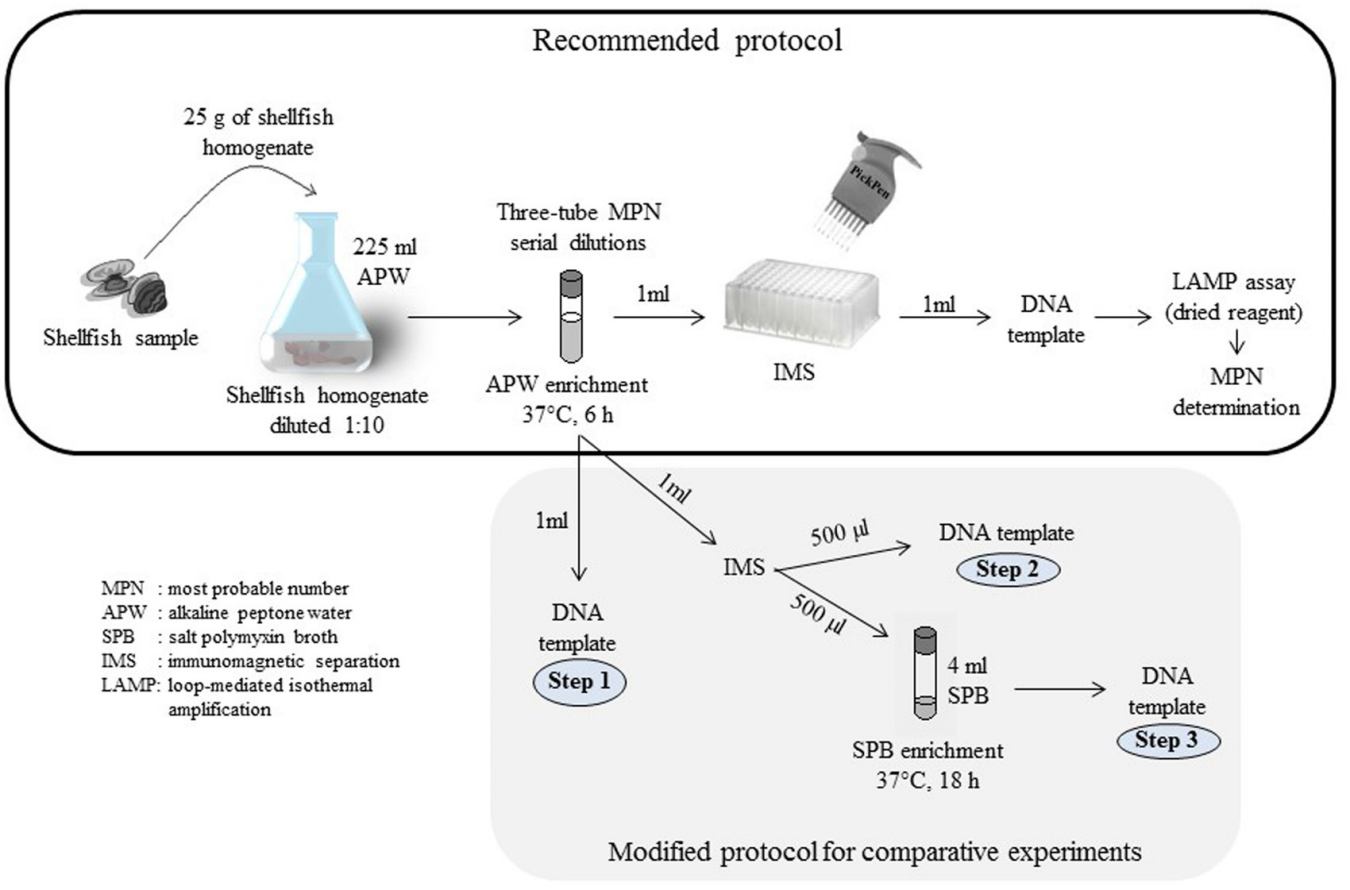
**Schematic representation of the strategy used to improve the method for quantitation of *tdh*^+^*Vibrio parahaemolyticus***.

To improve the MPN-IMS-LAMP previously reported (Tanaka et al., 2014), we evaluated the components of the protocol in two stages to propose the recommended procedure for quantitation of *tdh*^+^ *V. parahaemolyticus* and evaluated the recommended procedure as schematically shown in **Figure 2**.

### Stage I: Laboratory Evaluation of the Techniques

#### IMS: PickPen Operation Time

**FIGURE 3:**
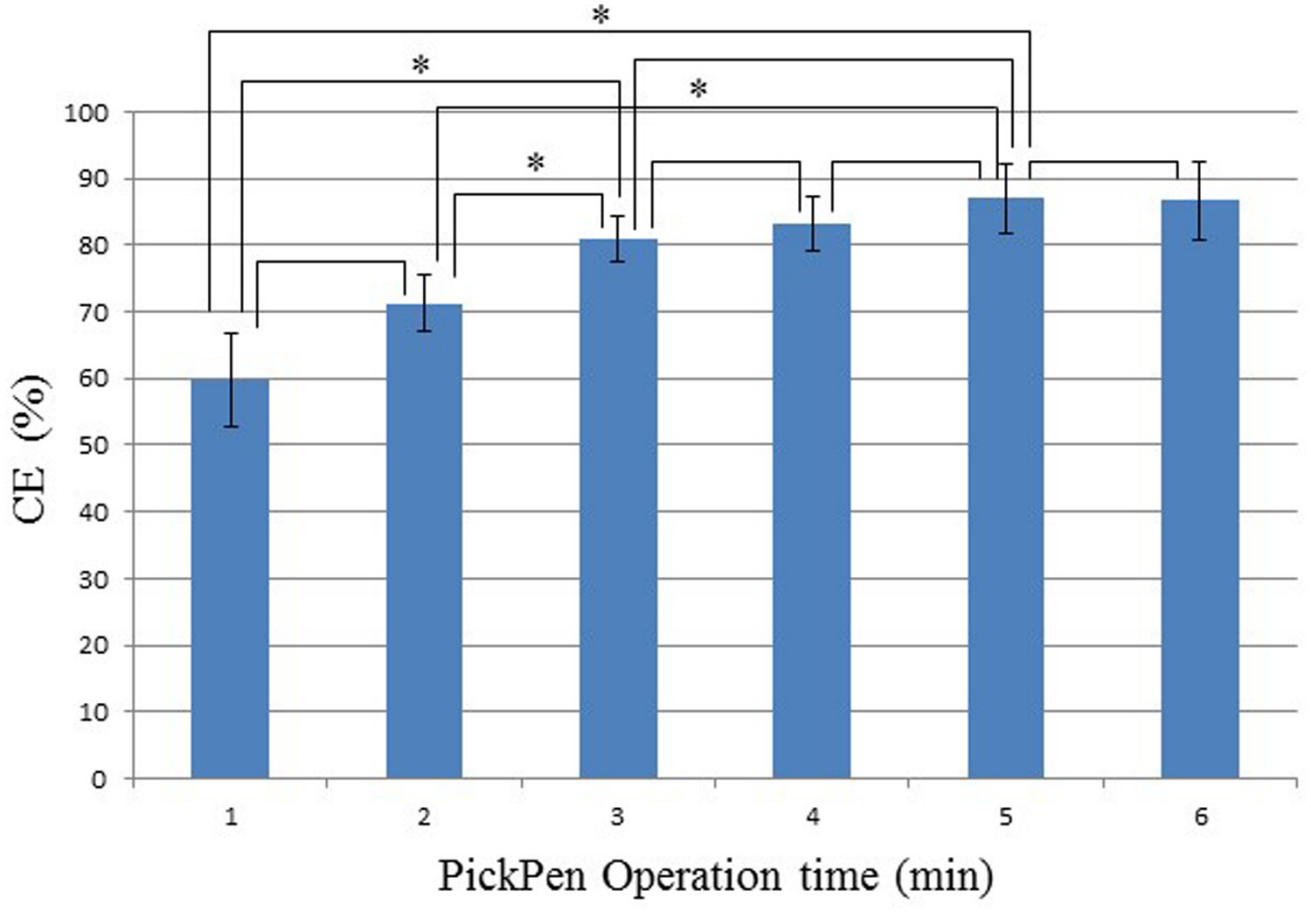
**Effect of PickPen Operation time on the capture efficiency (CE) of *V. parahaemolyticus***. CE (%) = (Co-Ca)/Co x 100%, where Co is the total CFU/ml present in the sample, and Ca is the CFU/ml not bound to IMB (Zeng et al., 2014). The pairs of the samples varying in PickPen Operation time that were compared for the difference in CE (%) using the Student’s *t*-test are indicated; ^*^pairs showing a significant difference (*p* = 0.05).

As explained in the introduction, we noticed loss of IMB during PickPen Operation suggesting improvement of this step. Therefore, we evaluated whether increasing the time during the washing step of IMB helped to minimize the loss of IMB and thus improve the CE of IMB. We used the student’s *t*-test to compare the different PickPen Operation times (1–6 min) and to evaluate the significance of increasing time (**Figure 3**). The CE value gradually increased with increase in the PickPen Operation time until it reached 5 min; in particular, the significant increase in CE was apparent after an PickPen Operation time of 3 min.

#### LAMP: Dried Reagent vs. Liquid Reagent

**FIGURE 4:**
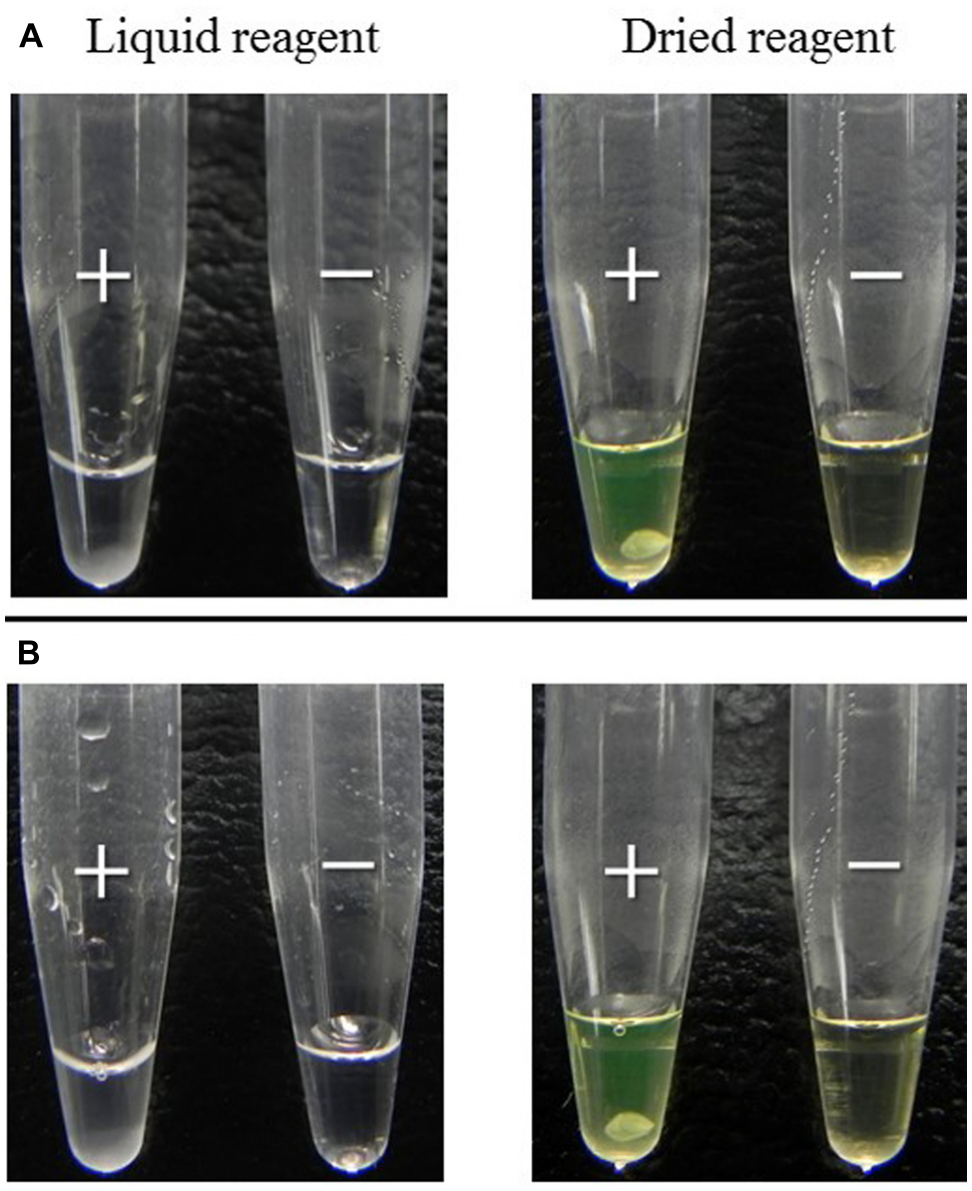
**Comparison of the LAMP results between liquid and dried reagents**. DNA template obtained using the boiling method from: **(A)** the pure culture of control strains examined for LAMP component at Stage I (**Figure 2**); **(B)** retail Thai shellfish samples examined at Stage III **Figure 2** by the recommended MPN-IMS-LAMP protocol **Figure 1**. Positive (+) and negative (-) LAMP reaction directly judged by eye.

To evaluate whether easy-to-use dried reagent can replace the standard liquid reagent we compared the two methods using 14 reference strains. The method using the liquid reagent or using dried reagent were equally sensitive and specific when the results were judged using the turbidimetric system (**Table 1**). In addition, when the product of the reaction was judged by eye, the results using the dried reagent were clearer, due to the color change of the rehydrated reagent from brown to green, than that using the liquid reagent (**Figure 4A**). Furthermore, it was more difficult to judge by eye the results of *trh* (*trh*1 and *trh*2) detection than *tdh* detection using the liquid reagent. Conversely, judgment of color by eye of the results for all these genes was equally easy when the dried reagent was used (data not shown). Further, the positive results in the *tdh* LAMP assays obtained after the dried LAMP reagent was exposed to different temperatures (30, 40, 50, and 60^∘^C), showed the dried reagent was equally stable at all of these temperatures during the examination period (15 days).

**Table 1:** Comparison of liquid reagent and dried reagent for detection of the *tdh*, *trh*1 and *trh*2 genes in *Vibrio parahaemolyticus* using the turbidimetric system.

Reference strains tested		No. of positive strains detected by the LAMP method targeting the gene(s)^*^ and LAMP reagent in liquid (L) or dried (D) form							
Genotype	No. of strains	*tdh*		*trh*1		*trh*2		*tdh*+*trh*2	
		L	D	L	D	L	D	L	D
*tdh*^+^	11	11	11	0	0	0	0	11	11
*trh*1^+^	1	0	0	1	1	0	0	0	0
*trh*2^+^	2	0	0	0	0	2	2	2	2

*^∗^Yamazaki et al. (2010).*

### Stage II: Evaluation of the Enrichment Procedure Using Shellfish

#### Change in the Enrichment Step for MPN Determination

We explored the possibility of finding a factor affecting the protocol in the enrichment step. We compared the MPN values after each of the three treatments (**Figure 1**, Steps 1–3) in the new protocol including the above modifications. Twenty-eight shellfish samples purchased in southern Thailand were examined for the MPN of *tdh*^+^ *V. parahaemolyticus* as described above (**Table 2**). The data clearly shows *tdh*^+^ *V. parahaemolyticus* is prevalent and the concentration is very high in these samples, with the log MPN/10 g value ranging from 1.0 to 5.4. Hard clams gave higher log MPN than the other two shellfish. Regardless of the kind of the shellfish, the values in Step 3 were lower than those of the other two steps. When the MPN values of Steps 1 and 2 are compared, three samples (**Table 2**, sample no. 8–10) were higher in Step 1, two samples (**Table 2**, sample no. 1 and 2) were higher for Step 2 and 23 samples were the same. The average MPN values for all samples between Steps 1 and 2 did not differ significantly.

**Table 2:** Comparison of the MPN values of *tdh*^+^ *V. parahaemolyticus* obtained at the three Steps of the modified protocol^∗^.

			log MPN/10 g		
Kind of shellfish	Sample designation	Date of examination	Step 1	Step 2	Step 3
Bloody clam	1	26-April	1.6	2.4†	1.6
(*Anadara granosa*)	2	22-April	2.0	2.6†	1.0
	3	25-April	2.4	2.4	1.4
	4	5-May	3.0	3.0	2.4
	5	6-May	3.0	3.0	2.6
	6	8-May	3.0	3.0	2.0
	7	12-May	3.4	3.4	2.6
	8	24-April	3.6†	3.0	2.0
	9	6-May	3.6†	3.4	2.6
	10	9-May	3.6†	3.4	2.6
	11	11-May	3.6	3.6	2.6
	12	8-May	3.6	3.6	3.4
		
		Average	3.03	3.07	2.23
		
Hard clam	13	23-April	2.4	2.4	2.0
(*Meretrix lusoria*)	14	28-April	3.4	3.4	3.0
	15	29-April	3.6	3.6	2.6
	16	25-April	3.6	3.6	3.0
	17	1-May	3.6	3.6	2.6
	18	5-May	4.0	4.0	3.0
	19	12-May	4.0	4.0	3.0
	20	7-May	4.6	4.6	3.6
	21	9-May	5.2	5.2	4.0
	22	6-May	5.2	5.2	4.4
	23	13-May	5.4	5.4	3.4
	24	24-April	5.4	5.4	3.4
		
		Average	4.2	4.2	3.2
		
Green mussel	25	22-April	2.4	2.4	2.0
(*Perna viridis*)	26	30-April	3.0	3.0	2.0
	27	13-May	4.0	4.0	2.9
	28	1-May	5.0	5.0	4.0
		
		Average	3.6	3.6	2.7
		
		**Total average**	**3.61**	**3.62**	**2.7**

*^∗^Explained in **Figure 2**. † MPN/10 g that differs between Steps 1 and 2.*

### Stage III: Evaluation of the Recommended MPN-IMS-LAMP

#### Sensitivity and MPN Accuracy Determination

Shellfish homogenates artificially contaminated with a reference strain (*tdh*^+^) at known concentrations were examined by the recommended protocol. Positive tubes in the three-tubes MPN format were 0-0-0, 3-0-0, and 3-3-0, for the homogenates contaminated at 0.1, 1.0 and 10.0 CFU/ml, respectively. The sensitivity of the recommended MPN-IMS-LAMP was therefore determined to be 1 CFU/ml. Based on the 3-tubes MPN format results, the MPN values obtained were <0.03 (range: <0.005–0.09) MPN/ml, 0.23 (range: 0.04–1.2) MPN/ml, and 2.4 (range: 0.36–13.0) MPN/ml, respectively.

#### Examination of Retail Shellfish in Thailand and Japan

Twenty-eight Thai samples were examined using liquid LAMP reagent in Stage II. Three (**Table 2**, sample designations 4, 21, and 25) of the 28 samples representing each shellfish group were also examined using dried LAMP reagent as in the new recommended protocol. The MPN values obtained at this stage were exactly the same, indicating the dried reagent had the same sensitivity as the liquid reagent as judged by the turbidimetric system. However, we confirmed that judgment by eye is easier when dried reagent is applied (**Figure 4B**).

Sixteen Japanese shellfish samples were also examined by the recommended new protocol (**Table 3**). The *tdh* gene was not detected in 14 of the 16 samples, with the MPN values being below the detection limit (<3 MPN/10 g). Two of the samples were positive for the *tdh* gene but the MPN values (15 and 23 MPN/10 g) were much lower than those obtained with Thai samples.

**Table 3:** Examination of the Japanese shellfish for the MPN of *tdh*^+^ *V. parahaemolyticus*.

Kind of shellfish	No. of samples	MPN/10 g	Origin and date of examination
Japanese littleneck (*Ruditapes philippinarum*)	11	<3.0	Shizuoka: 8-August (1), 4-September (1), 23-September (2)
			Kumamoto: 8-August (1), 4-September (2), 23-September (1)
			Chiba: 8-August (1), 4-September (1), 23-September (1)
	1	15.0	Kumamoto: 8-August
Hard clam (*Meretrix lusoria*)	3	<3.0	Ehime: 8-August (2), 4-September (1)
	1	23.0	Ehime: 23-September

*The numbers in the parentheses indicate the numbers of the samples examined.*

## Discussion

Our international research group attempted to develop an easy and sensitive method for quantitative detection of *tdh*^+^ *V. parahaemolyticus* in molluscan shellfish that can be applied in any part of the world; being practical and feasible even in resources-limited and/or tropical countries. We previously reported an MPN-IMS-LAMP method that was shown to be more sensitive than the MPN-PCR-based method (Hara-Kudo et al., 2003; Jones et al., 2012; Tanaka et al., 2014). However, a problem of under-estimation of the MPN values for some samples in a tropical environment was found as well as the technical practical inconvenience of the liquid LAMP reagent. In the current study, we improved the previously reported method using a series of technical modifications in the MPN and IMS components (**Figure 2**). In addition, we replaced the liquid reagent by the dried reagent in the LAMP component. As a result, much of the technical problems were solved (**Tables 2 and 3**; **Figure 4**).

While our study was in progress, a LAMP (targeting the *tlh* gene) and IMS (using nanoparticules targeting flagella) detection method for *V. parahaemolyticus* was reported (Zeng et al., 2014). However, CODEX 2011 recommends quantitative detection of pathogenic rather than total *V. parahaemolyticus* for risk assessment of *V. parahaemolyticus* in seafood (Food and Agriculture Organization World Health Organization of the United Nations [FAO/WHO], 2011). Along this line, our IMS method screens for clinically important *V. parahaemolyticus* by targeting all established K antigens and our LAMP method targets the *tdh* gene. Though, primer sets for *trh*1 and *trh*2 are available, variation in the *trh* gene sequence is widely observed in environmental strains is of major concern (Kishishita et al., 1992). A new *trh* LAMP primer set to overcome this issue is currently under development (Escalante-Maldonado and Nishibuchi, unpublished data).

The IMS component is an essential part of our protocol, therefore we needed to improve the efficiency of the IMS performance. *In vitro* experiments in Stage I confirmed that IMB incubation with constant shaking (Nou et al., 2006) for 30 min (Tanaka et al., 2014) enhances IMB-target association (data not shown). **Figure 3** shows that increasing the PickPen Operation time up to 3 min (1–3 and 2–3 min) increased CE value significantly. Increase in operation time after 3 min showed no significance difference in CE value where further extension of PickPen Operation time increases the workload. Taken together, we judged 3 min is the most effective PickPen Operation time. *In vivo* experiments conducted in Thailand employing the new protocol confirmed that these two modifications are a valuable contribution to the IMS performance (**Table 2**). The MPN values obtained after IMS application in this study are higher than the under-estimated MPN values reported in our previous study where similar shellfish samples were examined (Tanaka et al., 2014).

Another very valuable contribution to our recommended protocol is the application of the dried LAMP reagent. We utilized the dried reagent included in the commercially available kit for detection of two other pathogens. Results of the comparative experiments in Stage I indicate that both dried and liquid reagents are equally sensitive and specific for the LAMP assays for *V. parahaemolyticus* regardless of the primer set (**Table 1**). Evaluation of the stability of the dried reagent at different temperatures (30–60^∘^C) confirmed the dried reagent is very stable even at high-temperatures during the examination period, corroborating its utility even in tropical coastal areas of the world. Likewise, the dried reagent was proven useful because its transportation and storage do not necessarily require refrigerated or frozen conditions as confirmed during experiments in southern Thailand in Stage III.

Prevalence of infection by *tdh*^+^ *V. parahaemolyticus* was reported previously in southern Thailand. This prevalence is due to consumption of under-cooked molluscan shellfish, which is very common in southern Thailand (Laohaprertthisan et al., 2003). A risk assessment study conducted in southern Thailand reported low concentration of *tdh*^+^ *V. parahaemolyticus* in the bloody clam sold in the evening market (Yamamoto et al., 2008). However, pre-incubation of shellfish samples prior to examination may allow growth of *tdh*^+^ *V. parahaemolyticus* to a detectable level (Hara-Kudo et al., 2003; Yamamoto et al., 2008). Our previous study indicated that molluscan shellfish kept overnight and sold at the morning market contained relatively high levels of *tdh*^+^ *V. parahaemolyticus* (Tanaka et al., 2014). Under this condition, the under-estimation problem of the MPN values was raised. The current study examining similar shellfish samples solved the under-estimation problem. Comparative experiments in Stage II showed the problem is due at least in-part to the addition of the SPB enrichment step (**Figure 1**, Step 3). Among the MPN values observed at the three different steps, those of Step 3 were lower for all seafood samples examined (**Table 2**). SPB enrichment probably supported preferentially the growth of competing bacterial population rather than that of *tdh*^+^ *V. parahaemolyticus.*

The method yielding the highest MPN values is presumed to be most sensitive. We therefore compared the values obtained at Steps 1 and 2. The averages of the MPN values for these two steps were indistinguishable (**Table 2**). Difference in MPN values between Step 1 and Step 2 were observed only with five of 12 bloody clam samples. Two samples require special attention. They showed lower MPN values at Step 1 which were close to the detection limit. Application of IMS could avoid under-estimation of these values and assure the detection of the target.

## Conclusion

Modifications in the three components of the protocol (**Figure 2**) were critical to improve the MPN-IMS-LAMP. The under-estimation problem was solved by modifying the IMS and excluding the SPB from the enrichment step. Introduction of the dried LAMP reagent made the method quicker, easier and allows its use at high environmental temperatures. We therefore recommend the modified MPN-IMS-LAMP for detection and quantitation of *tdh*^+^ *V. parahaemolyticus* as a universal method useful in tropical, subtropical, and temperate zones of the world.

## Author Contributions

Conceived and designed the experiments: OE-M and MN. Performed the experiments and analyzed data: OE-M and AK. Technical assistance: WY, VV, and YN. Interpretated and wrote the paper: OE-M and MN.

## Conflict of Interest Statement

The authors declare that the research was conducted in the absence of any commercial or financial relationships that could be construed as a potential conflict of interest.
